# Morbidity and mortality of serious gastrointestinal complications after lung transplantation

**DOI:** 10.1186/s13019-019-0983-y

**Published:** 2019-10-28

**Authors:** Annette Zevallos-Villegas, Rodrigo Alonso-Moralejo, Félix Cambra, Ana Hermida-Anchuelo, Virginia Pérez-González, Pablo Gámez-García, Javier Sayas-Catalán, Alicia De Pablo- Gafas

**Affiliations:** 1 Department of Respiratory Medecine, Lung Transplant Unit, “12 de Octubre” University Hospital, “i + 12” Research Institute, Avda de Córdoba s/n, 28041 Madrid, Spain; 2 Department of General and Digestive Surgery, “12 de Octubre” University Hospital, Madrid, Spain; 3 Department of Anesthesiology, Lung Transplant Unit, “12 de Octubre” University Hospital, Madrid, Spain; 4 Department of Thoracic Surgery, Lung Transplant Unit, “12 de Octubre” University Hospital, Madrid, Spain

**Keywords:** Lung transplantation, Surgical complication, Gastrointestinal complications, Mortality

## Abstract

**Background:**

Gastrointestinal complications after lung transplatation are associated with an increased risk of morbidity and mortality. This study aims to describe severe gastrointestinal complications (SGC) after lung transplantation.

**Methods:**

We performed a prospective, observational study that included 136 lung transplant patients during a seven year period in a tertiary care universitary hospital. SGC were defined as any diagnosis related to the gastrointestinal or biliary tract leading to lower survival rates or an invasive therapeutic procedure. Early and late complications were defined as those occurring < 30 days and ≥ 30 days post-transplant. The survival function was calculated through the Kaplan-Meier estimator. Variables were analyzed using univariate and multivariate analysis. Statistical significance was defined as *p* < 0.05.

**Results:**

There were 17 (12.5%) SGC in 17 patients. Five were defined as early. Twelve patients (70.6%) required surgical treatment. Mortality was 52.9% (*n* = 9). Patients with SGC had a lower overall survival rate compared to those who did not (14 vs 28 months, *p* = 0.0099). The development of arrhythmias in the first 48 h of transplantation was a risk factor for gastrointestinal complications (*p* = 0.0326).

**Conclusions:**

SGC are common after lung transplantation and are associated with a considerable increase in morbidity-mortality. Early recognition is necessary to avoid delays in treatment, since a clear predictor has not been found in order to forecast this relevant comorbidity.

## Background

Lung transplantation has now become a standard treatment option in patients with end-stage lung disease. Each year the number of lung transplants increases significantly in the world. A total of 55,795 adult lung transplants have been performed through June 2016. Of these, 2273 (4.1%) were lung retransplantations.

Due to the growing experience of specialized centers, the average survival rate has increased to 5.8 years, reaching a 80% survival rate at year 1 and 54% at 5-years post-transplant [1] due to advances in surgical techniques and immunosuppressive therapy.

These improvements have decreased the main immediate complications of lung transplantation, especially extra-thoracic complications, of which gastrointestinal complications are of particular interest given their potential need for immediate surgical intervention [2].

Previous studies have shown that gastrointestinal complications are frequent in patients who have undergone lung transplantation and are an important source of postoperative morbidity and mortality. There are also reports that these complications may have an independent impact on patient survival [2–7]. Among them, early-onset (< 30 days) and severe complications have been associated with high mortality rates after lung transplantation [8, 9]. However, few studies have covered gastrointestinal complications in these patients.

The aim of the present study is to describe the frequency of severe gastrointestinal complications after lung transplantation, their impact on survival, as well as possible risk factors involved.

## Methods

We conducted a prospective, observational study that included all lung transplant patients from October 2008 to October 2015 at the “12 de Octubre” University Hospital. We collected demographic data, information related to the transplant, and occurrences of severe gastrointestinal complications up to the follow-up period. Retransplantation patients were excluded. Infectious causes were excluded.

When added to a transplant waiting list, interested patients signed an informed consent form authorizing the use of histological and clinical data from a biobank. This form was approved by the Institutional Review Board at the “12 de Octubre” University Hospital.

The type of complication, treatment and mortality were recorded. Complications were classified according to type of onset (early and late). All patients underwent basiliximab induction followed by maintenance therapy with three immunosuppressants: tacrolimus, microphenolate mofetil and corticosteroids most common regime according to the guidelines of the international society for heart and lung transplantation (ISHLT). Changes in immunosuppressant regimens were made due to nephrotoxicity, neurological and hematologic toxicity using alternatives such everolimus, cyclosporine and azatriopine.

Severe gastrointestinal complications were defined as any diagnosis related to the gastrointestinal or biliary tract leading to an important clinical repercussion for the patient that potentially endangers their survival or requires an invasive therapeutic procedure. Early and late complications were defined as those occurring < 30 days and > 30 days post-transplant.

A low cardiac output state after transplantation is defined as a syndrome evidenced by low cardiac output or cardiac index (cardiac index < 2 L/min/m^2^) requiring vasoactive drugs and conventional hemodynamic support the first 48 h of intensive care. The presence of arrhythmias after transplantation is defined as the existence of rhythm disorders during the first 48 h of intensive care, were included atrial fibrillation, atrial flutter, ventricular tachycardia, ventricular fibrillation and paroxysmal supraventricular tachycardia.

We used extracorporeal circulation (ECC) planned in patients with pulmonary arterial hypertension moderate-severe pre-transplant, this is urgent when occuring desaturation or pulmonary arterial hypertension during the surgery. We use extracorporeal membrane oxygenation (ECMO) pre and post transplant in cases hypoxemic respiratory failure refractory (venovenous) and cardiac or circulatory failure (venoarterial).

We analyzed overall survival by subgroups and variables were chosen based on previous studies [9, 10]. Demographic variables and comorbidities previous that might influence or be a risk factor for severe gastrointestinal complication. As well as variables can be imply a hemodynamic instabilit, transfusions, arrhythmias and mechanical cardiopulmonary support (ECMO and ECC). All potential risk factors tested were compared with the patients who experienced complications against those who did not.

The information was obtained from the hospital database of lung transplants and medical records. The data was collected in a Microsoft® Access database and stored according to regulations that govern clinical trials data. Continuous data were tested for normality (Shapiro–Wilks and Kolmogorov–Smirnov testing). Categorical variables were analyzed using Fisher’s exact test. Continuous variables were analyzed using t-test or ANOVA. For non-normal data the Mann-Whitney U and Kruskall-Wallis test were used. Potentially influential variables were analyzed using multivariate analysis and chi-square test. Statistical significance was defined as *p* < 0.05. The survival function was calculated through the Kaplan-Meier estimator. The comparison of the survival functions between groups was performed using log-rank test.

## Results

During the study period, a total of 136 patients underwent lung transplantation Of these, 87 (64%) were bilateral and 49 (36%) were unilateral. 58 patients (42.6%) required ECC and 10 patients (7.3%) ECMO. The mean age at the time of transplantation was 52.8 years (range 14–70), with 75 (55.1%) being men. The main indications of transplantation are listed in Table 1.

**Table 1:** Indications for Lung Transplantation (*N* = 136)

COPD	45 (33.1%)
IPF/UIP	37 (27.2%)
Primary pulmonary arterial hypertension	23 (16.9%)
Cystic fibrosis	11 (8.1%)
Other DILD	7 (5.1%)
α_1_ deficiency	4 (2.9%)
HX/LAM	4 (2.9%)
Other causes	5 (3.7%)

*COPD* chronic obstructive pulmonary disease, *IPF* idiopathic pulmonary fibrosis, *UIP* usual interstitial pneumonia, *DILD* diffuse interstitial lung disease, *HX* histiocytosis X. *LAM* lymphangioleiomyomatosis

Of 17 patients (12.5%) with SGC, ten were men. Three patients had pre-transplant gastrointestinal complications. These were one acute cholangitis resolved with medical treatment, one acute appendicitis and one intestinal perforation resolved with surgery. 12 patients received bilateral transplants and five received unilateral transplants. Patient demographics and transplant data are described in Table 2.

**Table 2:** Demographics of Patients With Severe Gastrointestinal Complications (*N* = 17)

Mean age at transplantation (years)	56.2 (range 38-66)
Male gender	10 (58.8%)
Primary pulmonary hypertension	5 (29.4%)
IPF/UIP	5 (29.4%)
COPD	4 (23.5%)
Other indications	3 (17.7%)
Bilateral transplant	12 (70.6%)
Extracorporeal Circulation	9 (52.9%)
Average EC time (minutes)	278.2 ± 68.8
Urgent	2 (22.2%)
ECMO	3 (17.6%)
Acute lung transplant rejection	4 (23.5%)

*COPD* chronic obstructive pulmonary disease, *IPF* idiopathic pulmonary fibrosis, *UIP* usual interstitial pneumonia, *EC* extracorporeal circulation, *ECMO* extracorporeal membrane oxygenation

Severe gastrointestinal complications included biliary pathology (*n* = 4), colonic pneumatosis (*n* = 3), intestinal perforation (*n* = 3), upper gastrointestinal bleeding (*n* = 2), low gastrointestinal bleeding Obstructive (*n* = 2) and acute pancreatitis (*n* = 1). Regarding the time of presentation, the majority was late with a median 95 days (interquartile range 32–213). Five severe gastrointestinal complications occurred early: all cases of intestinal perforation (*n* = 3), obstructive cause (*n* = 1), and low gastrointestinal bleeding (*n* = 1) (Table 3).

**Table 3:** Description of Patients With Severe Gastrointestinal Complications *(n* = 17)

Complications	N	Timing		Operative management		Mortality
		Early	Late	Yes	No	
Biliar pathology: Acute Cholecystitis Papillitis due to cholelithiasis	4 (23.5%) 3 1	0	4	4	0	1
Paralytic ileus Small bowel obstruction Large bowel obstruction	2 (11.7%) 1 1	1	1	2	0	2
Gastrointestinal bleeding Upper: Peptic ulcer AGML Lower: Polys	4 (23.5%) 1 1 2	1	3	3	1	3
Perforations Diverticulitis Peritonitis	3 (17.6) 2 1	3	0	3	0	2
Pneumatosis coli	3 (17.6)	0	3		3	1

*AGML* Acute gastric mucosal lesions

Twelve patients (70.6%) required surgical treatment, 5 of them in an emergency indication (41.6%) due to intestinal resection (*n* = 2), discharge colostomy (n = 2) and complicated peptic ulcer (n = 1). Mortality in patients with severe gastrointestinal complication was 52.9% (*n* = 9), with gastrointestinal complications being a direct cause of death in 17.6% (*n* = 3).

**Fig. 1:**
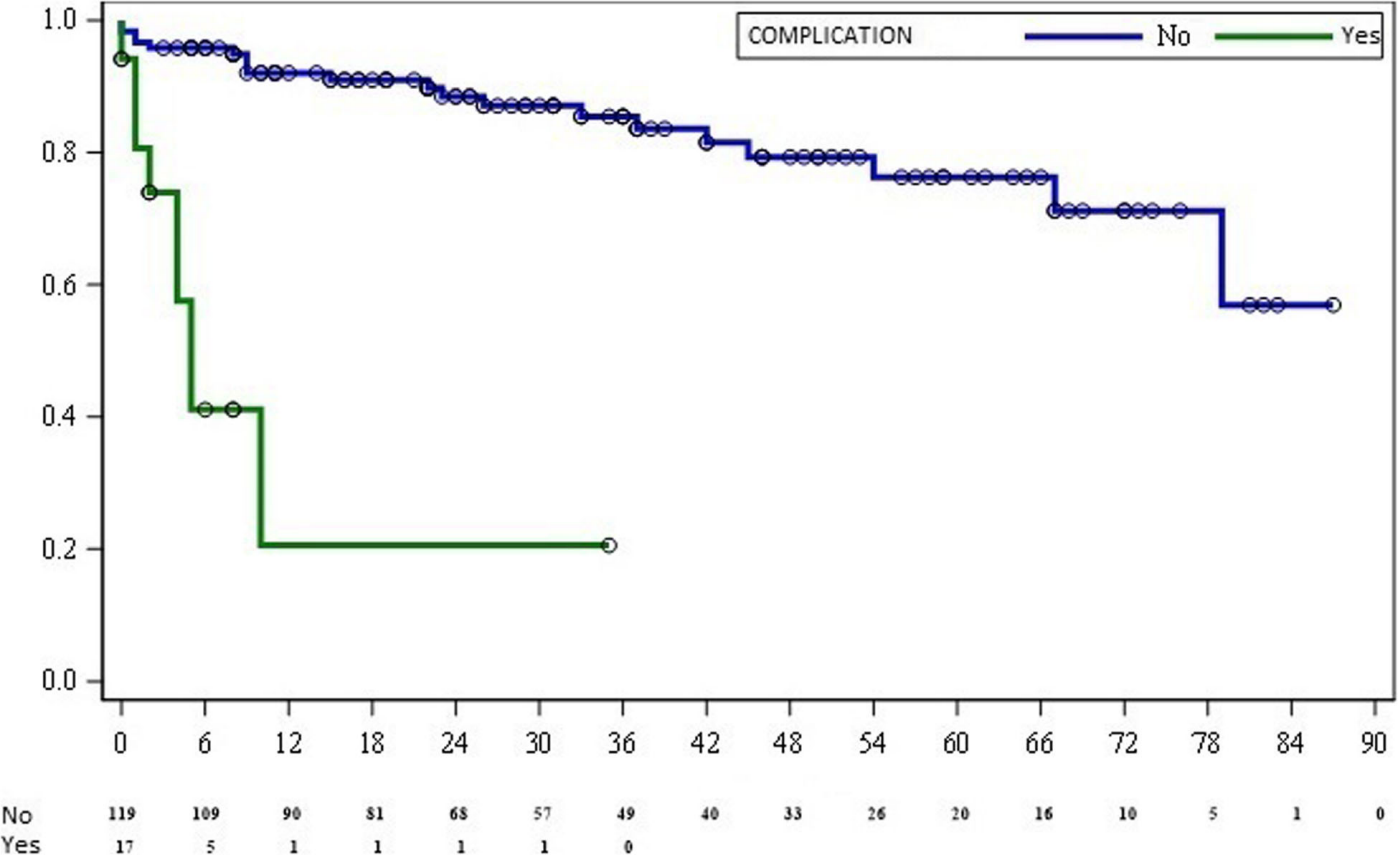
Kaplan-Meier survival analysis of the conformal groups: patients with severe gastrointestinal complications (n = 17) and patients without severe gastrointestinal complications (*n* = 119) (*p* = 0.0099)

Patients who had with severe gastrointestinal complications had a lower survival rate than patients without complications (14 months-95%CI 10.2–26.5 vs 28 months 95% CI 28.7–37.1, *p* = 0.0099) (Fig. 1).

Patients who presented early severe gastrointestinal complications (< 30 days) had a lower cumulative survival rate with a median of 4 months (95% CI 0–34.2) compared to late complications (> 30 days) with a median of 17 months (95% CI 9.9–30.3), *p* = 0.2448. The difference was not statistically significant. The presence of low cardiac output state and arrhythmias in the 48 h of the lung transplant had a significant influence on overall survival (*p* = 0.0194) and (*p* = 0.0240) statistically significant.

Univariate analysis identified that the development of arrhythmias in the first 48 h of transplantation is a risk factor for severe gastrointestinal complications. Patients who had arrhythmias developed significantly more severe gastrointestinal complications than those who did not (*p* = 0.0326). In the multivaririate analysis the variable was not significant (Table 4).

**Table 4:** Risk Factors for Severe Gastrointestinal Complications

	Gastrointestinal Complication		
Variable	Percentage		*P*-value
Sex			
Male	58.8%		0.7446
Arterial hypertension	11.8%		0.8311
Diabetes Mellitus	17.6%		0.1239
Dyslipidemia	11.8%		0.9176
Etiology			0.5538
Group 1	23.5%		
Group 2	29.4%		
Group 3	0%		
Group 4	29.4%		
Group 5	17.6%		
Bilateral lung transplant	70.6%		0.5435
Extracorporeal circulation	52.9%		0.4319
Extracorporeal membrane oxygenation	17.6%		0.1239
Tacrolimus	88.2%		0.4515
Mycophenolate	88.2%		0.4514
Azathioprine	5.9%		0.2096
Cyclosporine	11.8%		0.4964
Red blood cells transfusion	52.9%		0.543
Plasma transfusion	35.3%		0.2668
Platelet transfusion	41.2%		0.1561
Arrhythmias within 48 hours of transplantation	41.2%		0.0326
Hemodynamic instability over 48 hours	41.2%		0.8946
Low cardiac output 48 hours post-transplant	23.5%		0.0641
	Median		
Age	55.5		0.5778
Time Ischemia (minutes) > 400	290.7		0.1333
Multivariate analysis			
	OR	95% CI	*P*-value
Arrhythmias within 48 hours of transplantation	0.472	[0.141-1.586]	0.2248
ECMO	0.346	[0.064-1.883]	0.2196
Diabetes Mellitus	0.249	[0.047-1.321]	0.1424
Azathioprine	4.771	[0.524-43.460]	0.1656
Time Ischemia (minutes) > 400	0.927	[0.274-3.141]	0.1404

Group 1: Idiopathic Pulmonary Fibrosis and usual interticial pneumonia. Group 2: Chronic Obstructive Disease and Alpha 1 Antitrypsin Deficiency. Group 3: Cystic fibrosis and bronchiectasis. Group 4: Primary arterial hypertension. Group 5: Other etiologies

## Discussion

Gastrointestinal complications after lung transplantion are not uncommon. In the literature it is reported to occur between 21 and 62% [3, 4, 6, 7, 10] of the time, depending on the classification, definition, population studied or according to their severity. However, all the studies agree that these complications are an important cause of morbidity and mortality, with early and severe onset associated with higher mortality [10].

There are few published articles about major or severe gastrointestinal complications after lung transplantation. A previous study reports that 40.5% (*n* = 83) of major complications are found in 205 lung transplant patients, with gastrointestinal factors being the direct cause of death in 4.8% (*n* = 4) [10]. Our study found a lower percentage of severe gastrointestinal complications (*n* = 17, 12.5%) in 136 lung transplant patients, with gastrointestinal factors being the direct cause of death in three patients, which represents a high mortality rate (17.6%). These differences in the incidences with other series are due to the fact that there is a great variety in classifying the gastrointestinal complications that include non-surgical gastrointestinal pathology and infections among others. However, when we evaluate the mortality, it is similar because it is the surgical abdominal pathology that will have the greatest impact.

More than half of the patients with severe gastrointestinal complications (n = 12) required surgery and 5 of them were urgent cases, which shows the impact of the complications during the post-transplant period. Some studies reveal an increase in the mortality rate from 9 to 35% after emergency gastrointestinal surgery following a lung transplant [4, 8, 11].

This clearly affects the overall survival of the lung transplant recipient. Survival was significantly reduced in patients who had severe gastrointestinal complications compared with those who did not, with similar results found in the literature [4, 5, 10].

The timing of the complication was not associated with high mortality, as early presentation of gastrointestinal complications (< 30 days) did not significantly differ in survival rate in comparison with late presentation (> 30 days). However, the types of complications found between groups were different: in the early complications group, bowel perforation was more frecquent and led to higher mortality.

There are few studies about the potential risk factors involved in the development of severe gastrointestinal complications [9, 10, 12]. Age and bilateral lung transplant (vs unilateral) have been described as risk factors associated with developing severe gastrointestinal complications [9, 10]. This association is explained by hypoxia since bilateral transplantation is associated with a longer ischemic time, longer procedure and decreased oxygenation with an increased risk of primary graft failure. In our study, these variables were not significant, most likely due to sample size and due to different population.

On the other hand, the main indications for lung transplantation include cystic fibrosis and chronic obstructive pulmonary disease (COPD), which are usually associated with higher rates of gastrointestinal complications [3, 9]. In our study there were no significant differences between the indications leading to lung transplantation and the development of severe gastrointestinal complications.

The immunosuppression of transplant recipients plays a major factor in the development of severe gastrointestinal complications. Cholecystitis and diverticulitis have been found more frequently in patients with severe immunosuppression compared to the general population [13, 14, 15]. We did not find significant differences between differents patterns of inmunosupression.

The presence of circulatory mechanical support is clearly associated with the development of gastrointestinal complications. One study found that 33% of patients experienced complications, with *Clostridium difficile* infections, digestive hemorrhage, ischemia and intestinal perforation requiring surgical intervention, the majority resulting in high mortality rate [16]. Similar to other case series [10], we analyzed ECC and ECMO, which were not found to be statistically significant.

Other studies have shown that the incidence of peroperative myocardial infarction and low postoperative cardiac output requiring massive use of vasopressor substances were significantly higher in patients who subsequently developed gastrointestinal complications after cardiac surgery [17].

In our study, the presence of low cardiac output and arrhythmias in the first 48 h after surgery had an negative impact on the overall survival of the patients and when determining its relationship with severe digestive complications, only the presence of arrhythmias was statistically significant in univariate analysis. The reason for this association has not been documented before in this type of surgery. However, it is plausible because after a lung transplant, hemodynamic instability usually follows, leading to intestinal hypoperfusion and arrhythmias, which could lead to microinfarcts and abdominal visceral ischemia. However, in the multivariate analysis the presence of arrhythmias was not associated as independent risk factor for developing severe gastrointestinal complications, probably is due to small sample size. Also one possible reason is that the development of GI complications is related to several aetiologies and precipitating factors, making it difficult to isolate one causal factor. In other surgeries the need for transfusions has a negative impact on survival [18–20]. Our study found no difference in survival rate nor in developing digestive complications.

It is critical to mention that only three of the patients died from the gastrointestinal complications, but when severe enough, these complications weaken the patient by exposing them to a second infectious complication, malnutrition or a condition that leads to death.

Given these results, it is important to obtain an early diagnosis of the gastrointestinal complications by meticulously addressing all gastrointestinal complaints by doing a daily physical exam and early radiological tests in order to not delay treatment. Further we would propose to perform an early treatment of arrhythmias.

This study has some methodological limitations. It is a prospective, observational study involving a single hospital. However, the literature is very limited information and inconclusive. The strength of this study is that there are no losses in follow-up, and the data collection is prospective and verified by a multidisciplinary team. The study has a follow-up period of 7 years, during which time there were and not are serious complications reported, resulting in a variable with sustained impact and little bias.

## Conclusions

Severe gastrointestinal complications are frequent in the lung transplant recipient and are associated high morbidity with a negative impact on overall survival.

It has not been possible to identify other factors involved in the development of severe gastrointestinal complications other than the development of arrhythmias within 48 h.

Future studies should include records from multiple centers in order to obtain a reproducible predictor model.

## Data Availability

The datasets used and/or analyzed during the current study are available from the corresponding author on reasonable request.
